# Novel Approaches to Surfactant Administration

**DOI:** 10.1155/2012/278483

**Published:** 2012-12-03

**Authors:** Samir Gupta, Steven M. Donn

**Affiliations:** ^1^University Hospital of North Tees and Durham University, Hardwick Road, Stockton-on-Tees, Cleveland, OH TS19 8PE, UK; ^2^Division of Neonatal-Perinatal Medicine, C. S. Mott Children's Hospital, University of Michigan Health System, Ann Arbor, MI 48109-4254, USA

## Abstract

Surfactant replacement therapy has been the mainstay of treatment for preterm infants with respiratory distress syndrome for more than twenty years. For the most part, surfactant is administered intratracheally, followed by mechanical ventilation. In recent years, the growing interest in noninvasive ventilation has led to novel approaches of administration. This paper will review these techniques and the associated clinical evidence.

## 1. Introduction

Respiratory distress syndrome (RDS) is the most common disease entity of premature infants. It is characterized by surfactant deficiency, immature airways, and lung parenchyma. With advances in the perinatal management, particularly antenatal corticosteroid therapy administered to the parturient, surfactant-deficient lung disease is now more prevalent among infants less than 29 weeks' gestation.

Surfactant is composed of phospholipids and associated proteins, produced by the type II pneumocytes that line the alveoli and smallest bronchioles. It reduces surface tension and stabilizes the air-liquid interface at the alveoli, thereby contributing to improvement in pulmonary compliance. While surfactant is necessary for normal lung function, adequate surfactant is not sufficient to assure normal gas exchange in the preterm infant. There are no simple ways to separate surfactant deficiency from other aspects of lung development, such as airway development, alveolarization, and the development of the pulmonary vasculature in the preterm infant. Babies born prematurely could be deficient in surfactant and also have underlying lung hypoplasia. Airway development also differs between infants of comparable gestational ages, as evidenced by susceptibility to the development of pulmonary interstitial emphysema. Nevertheless, surfactant treatment for RDS has been shown to dramatically improve survival of preterm infants.

The effects of surfactant therapy on RDS can be divided into pulmonary, cardiac, and radiologic. The immediate pulmonary effects include rapid improvement in oxygenation accompanied by increasing functional residual capacity, followed by a variable increase in lung compliance. The effects of surfactant administration on pulmonary artery pressure and pulmonary blood flow are not conclusive. Some studies suggest no changes in pulmonary flow after surfactant administration, while others suggest an increase in ductal shunt velocity and increased pulmonary blood flow. The radiologic changes reflect the recruitment of lung volume and decrease in atelectasis after surfactant treatment.

Administration of exogenous surfactant is the established treatment for RDS. It is the most widely studied drug in the last 25 years. The body of literature suggests that early or “prophylactic” administration of surfactant is more beneficial than late (rescue) therapy [1]. This has been a standard practice, and premature babies at risk of RDS often receive prophylactic surfactant in the delivery room during their initial stabilization. However, this approach is invasive, because it requires endotracheal intubation for administering the surfactant.

The complications of surfactant administration, which include bradycardia, hypoxia, and hypotension, and interest in noninvasive respiratory support, have highlighted the need to explore alternative forms of surfactant replacement therapy. One study assessed the intubation times and number of attempts between neonatal consultants, neonatal fellows, and pediatric residents in Australia. The findings reflected the relationship between neonatal experience and ease of intubation [2]. Secondly, with the results of recent randomized clinical trials, many clinicians prefer to stabilize babies using noninvasive respiratory support initially without giving surfactant [3]. A modified approach, referred to as INSURE, requires endotracheal intubation, administration of surfactant, followed by rapid extubation to noninvasive support [4]. It still entails the attendant risks of intubation.

Reports of noninvasive approaches to stabilization, using early CPAP [5, 6], renewed interest among clinicians and questioned the need for routine surfactant administration. These observational data suggested significantly less bronchopulmonary dysplasia at one center that used much less mechanical ventilation. During the same period, Verder et al. [4] tested a novel approach, INSURE (intubation, surfactant administration, and extubation). This technique provides the benefits of surfactant administration but also eliminates continued mechanical ventilation. This approach, however, still requires skills for intubation and has the potential for trauma to the glottis and airway during intubation as well as the risks of surfactant administration enumerated above. 

Over the last decade, randomized controlled trials have enrolled over 2500 infants to compare CPAP versus intubation and intermittent positive pressure ventilation (IPPV) at birth. Some trials (VON trial, IFDAS) also included INSURE as a third arm. Unfortunately, they reported no differences in the incidence of BPD or associated complications of prematurity [3]. 

With the uncertainty of initial management of these vulnerable premature infants, mechanical ventilation remains the “default” respiratory support. Some infants with mild surfactant deficiency may be managed without mechanical ventilation and surfactant administration for first few days, but the clinical problem is how to identify them. Borderline babies, who might do well initially, often develop signs of RDS over the next couple of days and may have a more difficult course because of delayed surfactant administration.

As mentioned above, intubation of the trachea can be hazardous and is usually undertaken after premedication, which may contribute to respiratory depression and a delay in extubation even after surfactant is administered. To incorporate the advantages of surfactant and to limit complications of endotracheal intubation, clinicians have been exploring other methods of surfactant administration, including delivery of surfactant via the upper airway or minimizing injury while administering intratracheal surfactant. Several techniques, collectively labeled “minimally invasive surfactant therapy” (MIST), have been described in which surfactant is delivered without tracheal intubation. These potential strategies include the following:

1. intra-amniotic instillation,
2. pharyngeal instillation,
3. administration via laryngeal mask airway,
4. administration via thin endotracheal catheter without IPPV,
5. aerosolized/nebulized surfactant administration in spontaneously breathing infants.

## 2. Intra-Amniotic Instillation of Surfactant

There is only one feasibility report describing endoscopic delivery of surfactant directly to the fetus during active preterm labor. Using this technique, Petrikovsky et al. [7] introduced a gas-sterilized intraoperative fiberscope through the cervical canal into the amniotic cavity after spontaneous rupture of membranes during preterm labor. Using this approach the investigators injected surfactant into the mouths of 3 preterm fetuses through a catheter placed through the biopsy channel of the fiberscope. They reported no complications but suggested the need for further prospective studies to confirm the safety and efficacy of this method. Thus far, it has not been incorporated into clinical practice.

## 3. Pharyngeal Instillation of Surfactant

Babies born at term normally initiate respirations by first inspiring air and then closing the glottis while attempting to exhale. This creates a significant positive transpulmonary pressure and presumably forces fetal lung fluid into the interstitium of the lung [8]. It is likely that this process results in establishment of an air-fluid interface in the alveolus with deposition of surfactant from the fetal lung fluid. However, in the preterm lung, where surfactant is deficient, a similar positive pressure may result in histologic disruption of alveolar integrity [9], release of cytokines [10], leakage of serum proteins [11], and inactivation of both endogenous surfactant and any exogenously administered surfactant [12]. The pharyngeal instillation of surfactant before delivery has the potential to replicate the physiologic process. While the chest remains compressed in the birth canal, fetal lung fluid can be suctioned from the upper airway and replaced with a surfactant-containing solution. Then, as the chest expands, the baby is stimulated to aspirate the surfactant-containing solution providing surfactant at the advancing air-fluid interface. This process can be further facilitated by the application of mask CPAP.

Utilizing this approach, the initial report of pharyngeal instillation of surfactant was published in 2004 [13]. Twenty-three infants (560 to 1804 grams) born between 27 and 30 weeks' gestation had surfactant (Infasurf) administered within the nasopharynx before delivery of the shoulders after suctioning of the nasopharynx. Newborns received CPAP at 10 cm H_2_O by mask as they initiated breathing, and this was continued at 6 cm H_2_O for at least 48 hours. The investigators reported the technique to be relatively safe and simple to accomplish during vaginal deliveries. Unfortunately, this approach requires a cephalic delivery and a spontaneously breathing infant. Cesarean section, malpresentation (breech or transverse), or perinatal compromise limit the application of this approach. A Cochrane review did not find any articles comparing this approach to no treatment or treatment with intubation and surfactant [14].

## 4. Administering via Laryngeal Mask Airway (LMA)

The LMA is a supraglottic device consisting of a curved plastic tube with an elliptical inflatable mask that is inserted blindly into the posterior pharynx of the baby. The mask may be inflated in the hypopharynx to create an airtight seal around the upper esophagus. It offers the possibility of rapidly establishing effective ventilation and access to the airway without the need for tracheal intubation, even when performed by relatively inexperienced personnel. There are different types of LMAs available (Classic; ProSeal; i-Gel; PAX press; CobraPLA). 

A protocol for LMA surfactant administration suggested by Trevisanuto [15] involves positioning the LMA, followed by instilling the surfactant in two to four aliquots via the LMA. Each aliquot is usually followed by brief IPPV until the surfactant disappears from the LMA. Once the surfactant aliquots have been completely administered, the LMA is removed and the baby is placed on CPAP for subsequent management.

There are no reported studies of prophylactic or early LMA surfactant administration [16]. One small study reported a comparison of late rescue LMA administration of surfactant versus no surfactant. This study enrolled 26 preterm infants ≥1200 g with RDS who required CPAP. LMA surfactant administration resulted in a reduction in the mean FiO_2_ required to maintain pulse oximetry between 88% and 92% for 12 hours after the intervention. No significant differences in subsequent mechanical ventilation, pneumothorax, days of intermittent positive airway pressure (IPPV), and days of IPPV or oxygen were reported [17]. 

Possible adverse effects of LMA surfactant administration include hypoxia and bradycardia during administration, laryngospasm, and malposition of the LMA, with potential effects on the newborn [18]. The limitations of surfactant administration using LMA are related to the nonavailability of smaller LMA sizes for use in extremely premature infants [18]. The technique is relatively simple and seems promising, but well-designed studies are needed to confer safety and efficacy.

## 5. Administration via Thin Endotracheal Catheter/Feeding Tube without IPPV

This method of surfactant administration delivers exogenous surfactant using a thin intravascular catheter or feeding tube inserted below the vocal cords. It is classified as a “MIST” technique. Using Magill forceps, a nasogastric tube is inserted into the trachea under direct laryngoscopic visualization of the vocal cords during nasal CPAP therapy. After placement of the catheter, surfactant is administered over a period of 1–3 minutes, while the infant continues to receive nasal CPAP. The procedure was first described in a feasibility study including premature infants ≤27 weeks of gestation. In this observational study, the intervention data were compared to historical controls. Reduced mortality (11.9% versus 35.3%, *P* = 0.025) and a reduced rate of severe IVH (grade 2 or 3) in survivors (5.1% versus 31.8%, *P* = 0.01) were observed [19]. After the publication of these results, some German centers adopted this method and they conducted a retrospective analysis of data from 15 centers. A total of 1541 infants <31 weeks of gestation was analyzed [20]. One thousand two hundred and twenty-two infants received standard care, and 319 were treated with the new method. Although smaller (945 versus 1018 g, *P* < 0.001) and less mature (27.3 versus 27.9 weeks, *P* < 0.001), infants treated with the new method showed less death or BPD (13.3% versus 19.9%, *P* = 0.007) and less need for any respiratory support. The technical difficulties associated with this method include the use of a highly flexible feeding tube and the need to use Magill forceps to advance the tube tip into the trachea. The necessary skills set may limit more widespread application.

To overcome the limitations imposed by the flexible nasogastric tube, Dargaville et al. tested the MIST technique utilizing placement of a 16-gauge vascular catheter below the vocal cords without using Magill forceps or premedication. This study enrolled 11 infants 25–28 weeks' gestation requiring any CPAP pressure or FiO_2_, and 14 infants 29–34 weeks' gestation at CPAP pressure ≥7 cm H_2_O and FiO_2_  ≥0.35. In all cases, surfactant was successfully administered and CPAP was reestablished. Coughing (32%) and bradycardia (44%) were transiently noted and 44% received positive pressure inflations. There was a clear surfactant effect, with lower FiO_2_ after MIST (pre-MIST: 0.39 ± 0.092; after 4 hour: 0.26 ± 0.093; *P* < 0.01), and a modest reduction in CPAP pressure. Few adverse outcomes were reported: intubation within 72 h (*n* = 3), pneumothorax (*n* = 1), BPD (*n* = 3), and death (*n* = 1), all in the 25–28-week group. Favorable outcomes were reported in both gestation groups, with a trend towards reduction in intubation in the first 72 h in the 25–28-week infants compared to historical controls [21].

The main limitations of the MIST methods are the need for laryngoscopy and the use of Magill forceps. There is still concern about potential trauma from both the laryngoscope and the catheters. In active preterm infants, in particular, placement of the catheter without sedation may be difficult and potentially traumatic, as well as uncomfortable. Additionally, this technique utilized a Benevista gas jet valve to provide CPAP while administering surfactant. This facilitates dispersion of surfactant without IPPV using a high flow CPAP system. It is unclear whether this method will be effective when used with Bubble CPAP or Infant flow driver CPAP.

## 6. Aerosolized/Nebulized Surfactant Administration in Spontaneously Breathing Infants

Many believe that the noninvasive administration of an aerosolized or nebulized surfactant might represent the best of all possible worlds by sparing manipulation of the airway but being able to administer surfactant early in the course of RDS. Until recently, aerosolization has remained elusive. In order for the parent surfactant to be effective, four steps need to be accomplished. First, the surfactant needs to be aerosolized. The energy to do so may denature surfactant proteins. Second, the appropriate particle size needs to be achieved so that it does not “rain out” in the airway and is capable of penetrating deep into the lung. Third, the particles must be able to reaggregate at their site of action. Finally, the reaggregated surfactant has to regain and maintain its biological activity.

Use of nebulized surfactant seems to be the most sophisticated and minimally invasive technique. Several pilot trials have utilized this technique [22–26]. The majority of these trials used nasal CPAP delivery. One of the studies showed an improvement in (A-a) O_2_-gradient, Silverman score, and PaCO_2_  [22] and another study failed to demonstrate efficacy [23]. The studies are difficult to compare, as different surfactant preparations and different devices for nebulization and delivery were used, including jet nebulizers, ultrasonic nebulizers, and vibrating membrane nebulizers. The postnatal ages at application also varied between less than 30 minutes to less than 3 days of age.

Arzhavitina examined an *in vitro* model comparing six different nebulizers. They reported differences in the process of aerosol droplet generation between drugs with and without properties of surface activity and according to the type of nebulizer. They hypothesized that a vibrating membrane nebulizer is the best device for substances with surface activity, such as surfactant, as the residual volume in the device is minimal and the substance output is maximal [27].

The typical protocol for nebulized surfactant administration involves the use of an aerosol generator with surfactant administered by a nasal CPAP system, using either a tight face mask or nasopharyngeal tube [24]. Multiple factors are reported to influence aerosol surfactant dose delivery, including patient weight or size, minute ventilation [28], aerosol flow and patient peak inspiratory flow, aerosol particle size (as large as possible to avoid potential exhalation, yet small enough to bypass the oropharynx) [26], type of aerosol generator used, and type of surfactant [29] (Table 1). Nebulized surfactant may reduce the need for endotracheal intubation and is well tolerated [22, 24], apart from transient oxygen desaturation during dosing. There are no trials comparing the efficacy of nebulized surfactant delivery in premature infants compared to the standard approach or other delivery methods. Further refinements may, however, make it an attractive technique for future consideration.

## 7. Summary

The current evidence regarding noninvasive surfactant delivery techniques in premature infants is limited to pilot data and feasibility studies. This is further complicated by varying delivery methods and nonavailability of smaller devices for use in very preterm infants. With the growing interest in noninvasive respiratory support techniques, until conclusive data on superiority of approach is documented, the gold standard of respiratory support is endotracheal intubation, administration of surfactant, and optimal mechanical ventilation. Data from clinical trials of the novel techniques will need to evaluate long-term respiratory and neurodevelopmental outcomes to prevent any untoward harm in vulnerable preterm infants and to assess the true cost effectiveness.

## Figures and Tables

**Table 1:** Factors influencing the success of aerosolized/nebulized surfactant.

(1) Patient weight	
(2) Minute ventilation	
(3) Aerosol flow	
(4) Patient peak inspiratory flow	
(5) Aerosol particle size	
(6) Type of aerosol generator	
(7) Type of surfactant	
